# A short comparative study on modified Duckworth-Lewis methods

**DOI:** 10.1371/journal.pone.0259423

**Published:** 2021-11-08

**Authors:** Muhammad Asif, Ali Ahmadian, Muhammad Azeem, Bruno Antonio Pansera

**Affiliations:** 1 Department of Statistics, University of Malakand, Lower Dir, Pakistan; 2 Institute of IR 4.0, The National University of Malaysia, UKM, Bangi, Selangor, Malaysia; 3 Department of Mathematics, Near East University, Nicosia, TRNC, Mersin, Turkey; 4 Department of Law, Economics and Human Sciences & Decision Lab, University Mediterranea of Reggio Calabria, Reggio Calabria, Italy; Sapienza University of Rome, ITALY

## Abstract

In this paper, the Duckworth-Lewis-Stern (DLS) and Duckworth-Lewis-McHale-Asif (DLMA) methods of revising targets for a team batting in second innings in an interrupted Limited Overs International Cricket (LOI), are examined for fairness. The work discusses four significant points: flexibility, intuition, simplicity, and goodness-of-fit of the two mentioned methods. The research findings have shown that the DLMA method is better in every aspect than the DLS method. Further, the data of 1764 ODI matches played during 2004–2021 to investigate the compatibility of the DLMA for high run-scoring One-Day International matches. The results show that DLMA is compatible to the situation of the well-above run-scoring situation.

## 1 Introduction

Limited Overs International (LOI) was introduced in 1970s. The International Cricket Council (ICC) has sought to have a quantitative method to revise target for a team batting in the second innings so that interrupted matches were concluded with a positive result. In the past, the ICC adopted the Run-Rate method, Most Productive Overs method, Duckworth-Lewis (DL) (Standard and Professional Editions) method. Presently, Duckworth-Lewis-Stern method, a modified form of the Duckworth and Lewis [1] proposed by Stern [2], is in operation by the ICC. The DL method is not only used to revise targets but had also used for statistical modelling in cricket (see, for example, Clarke and Allsopp [3], de Silva et al. [4], Bailey and Clarke [5], Asif and McHale [6, 7]).

Duckworth and Lewis [8] introduced a method of revising targets for a team batting in the second innings in an interrupted limited-overs match. The International Cricket Council (ICC) formally adopted the technique in 1999. This version of the DL method is known as Standard Edition. Since 1997, attempts had been made to improve the DL method. For example, Duckworth and Lewis [1] modified the method, known as then DL Professional Edition (DLPro), so that the method produced fairer adjusted targets in high-scoring interrupted games. Stern [9] proposed modification in the DLPro method. However, McHale and Asif [10] investigated and examined this modified form of DL method and concluded that the method proposed by Stern [9] violates the basic principles of the game.

In addition, McHale and Asif [10] proposed an improved version of the DL method by changing the functional form of the DL model completely. Recently, Stern [2] had made different modifications in the DLPro method known as Duckworth-Lewis-Stern (DLS) method. In this paper, the McHale and Asif [10] version of the DL method is referred to as DLMA. In the literature, alternative methods are also proposed, for example, Preston and Thomas [11], JVD of Jayadevan [12], Carter and Guthrie [13], a DL method for Twenty-20 International (T20I) of Bhattacharya et al. [14]. However, these methods had serious shortcomings (see Stern [2] and McHale and Asif [10] for details).

Stern [2] and McHale and Asif [10] reported that DLPro was not compatible with the modern-age runs scoring pattern. In DLMA, an alternative model for runs to be scored in the remaining innings as a function of overs left and wickets lost was proposed. However, Stern [2] only proposed modification in a parameter of the DLPro model called match factor, denoted by λ. Further, Stern [2] examined the fairness of the DLMA method and reported that only DLPro and DLMA do not violate any of the key principles of fairness as compared to the other alternative methods available in the literature. Asif and McHale [7] recently proposed a generalized non-linear forecasting model (GNLFM) for LOI cricket. The GNLFM is a generalization of the Duckworth-Lewis model. DLMA and DLPro models are the special cases of GNLFM.

Likewise, the issue of fairness in other sports extensively discussed in the academic literature. For instance, Wright [15, 16] provides a survey of sporting rules from an Operational Research (OR) perspective. Kendall and Lenten [17] is probably the first comprehensive review of sporting rules, which have led to unexpected consequences. Vaziri et al. [18] state properties of fair and comprehensive ranking methods in sports. Csatό [19, 20] analyze the fairness of ranking in Swiss system chess team tournaments. Guyon [21], Laliena and Lopez [22], and Cea et al. [23] study the fairness of the FIFA World Cup draw. Durán et al. [24] use integer programming to construct schedules for the South American Qualifiers to the FIFA World Cup that overcome the previous approach’s main drawbacks. Their proposal was unanimously approved by the South American Football Confederation (CONMEBOL) members and used in the qualifier tournament for the 2018 FIFA World Cup in Russia. See also Alarcón et al. [25]. The fairness of soccer penalty shootouts is extensively discussed in the academic literature, for instance, studies [26–32]. Several recent research papers investigate the incentive (in)compatibility of sports rules, that is, whether the teams are always interested in winning, which can be a basic criterion of fairness [33–39].

In this paper, the relative efficiency and viability of the DLS of Stern [2], and DLMA of McHale and Asif [10] are investigated. The flexibility, intuition, simplicity, and goodness-of-fit of the two methods are examined. Based on the research findings, it is concluded that the DLMA method is intuitively more flexible, simple, and has a better fit to the data as compared to the DLS method. In the next section, different versions of the Duckworth-Lewis method are discussed. In section 3, research findings and discussion on the relative efficiency of the DLMA and DLS are provided. Finally, future potential research directions and concluding remarks are given in section 4.

## 2 The Duckworth-Lewis methods

### a. Historical background (The Run-Rate method)

The Duckworth and Lewis (DL) [8] method was introduced in 1997 and adopted officially by the International Cricket Council (ICC) in 1999. The method was invented with the idea that each over (or ball) has different run scoring potential depending on how many overs left and number of wickets have already been lost. Prior to the DL method, various quantitative procedures were experimented by the ICC. For example, some of the commonly used methods were Run-Rate (RR) method, Highest Scoring Overs (HSO) method, Equivalent Point (EP) method, Word Cup 1996 (WC96) method, CLARK method, and Parabola (or PARAB) method. The fundamental flaws, anomalies, and shortcomings of these methods were comprehensively discussed in the literature [8, 11, 40]. Among these traditional methods, the RR method was mostly used in the Limited Overs International (LOI) cricket to revise targets for the team batting second in the interrupted matches.

**Fig 1:**
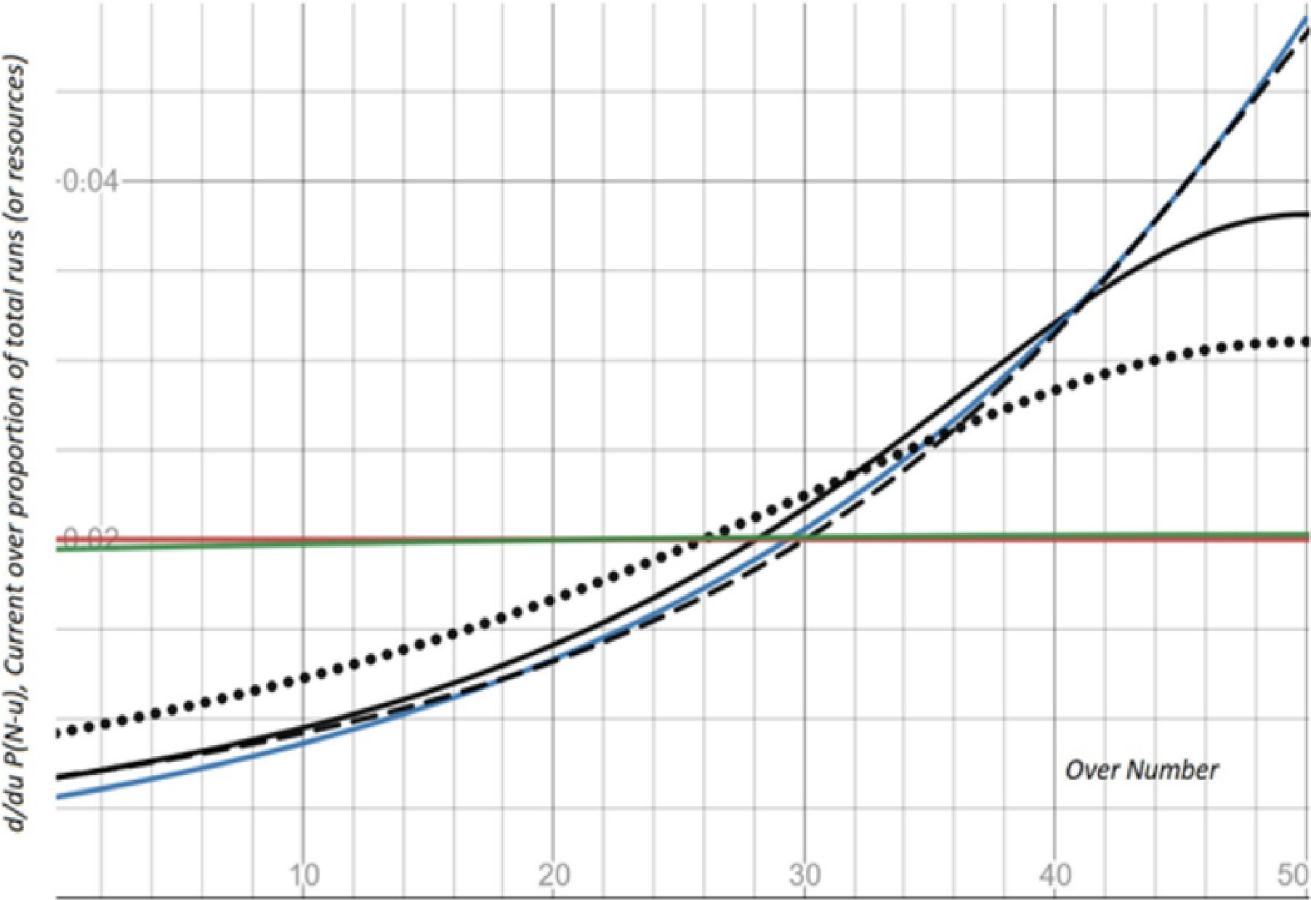
**Runs scoring pattern (over-by-over resources) of the DLMA (black and green curves,), DLS (blue curve) and RR (Red curve).** Vertical axis represents an expected proportion of runs (or resources value) to be scored in the current over for *N = 50*.

In the RR method, the average runs-per-over of each competing side is compared, and the team with the higher run rate is declared as the winner. The RR method is simple to implement but could unfairly favours either side, depending upon the situation. For example, other versions of this method, the maiden ignored run-rate method and the factored run-rate method, were also experimented [41]. However, the fundamental problems with the run rate based methods remained unresolved. The run-rate-based methods’ major flaw is non-flexibility, such as ignoring the wicket-lost effect and having uniform resources across all-overs (For detail, see [40]. The red curve in Fig 1 represents the current over resources as a function of the over number (or overs batted). It may be observed that the RR-based over-by-over resources remain constant irrespective of the over batted and wicket(s) lost.

### b. The Duckworth-Lewis professional edition

The fundamental ideal of the Duckworth-Lewis (DL) method is to estimate the resources available to each team in an innings. In an uninterrupted match, each team has 100% of its resources available. To estimate the resources available to a team, the Duckworth and Lewis [1] method use a model of the average runs remaining to be scored, *Z*. The Duckworth-Lewis model for the expected runs in the remaining overs *u* and given wickets *w* already been lost is given by

$$Z_{dl}(u,w|\lambda )=Z_{0}\lambda^{n(w)+1}F(w)\left[1-\exp\left\{-bu/\lambda^{n(w)}F(w)\right\}\right]$$ (1)

where *Z*_0_ is the asymptotic average runs with no wickets lost in hypothetically an infinite number of overs. *F*(*w*) is a positive decreasing step function with *F*(0) = 1. The parameter λ is known as the match factor. In matches with well above average targets, it scales down the rate parameter b, the relationship between *Z* and *u* for the given *w* tends to be more linear for λ > 1. Similarly, *n(w)* is a positive decreasing function with *n(0) = 5*. The ratio *P*_*dl*_(*u*,*w*) = *Z*(*u*,*w*)/*Z*(*N*,0) gives the average proportion of runs still to be scored in an innings with overs remaining *u* and wickets lost *w*. In innings *i* (*i* = 1,2), following *n*_*i*_ interruptions (the *j*^*th*^ interruption stops play when *u*_1*j*_ overs remain and *w*_*j*_ wickets have been lost, and play is resumed when *u*_2*j*_ overs remain), the available resources are given by $R_{i}=1-\sum_{j=1}^{n_{i}}\left\{P_{dl}(u_{1j},w_{j})-P_{dl}(u_{2j},w_{j})\right\}$. Duckworth and Lewis [1, 8] did not disclose the functional forms of *F(w)*, *n(w)*, estimated values, and the estimation procedure for their method, citing the reason for commercial confidentiality.

### c. The McHale-Asif version of the DL method

McHale and Asif [10] proposed a modified form of the DLPro method, referred to as the Duckworth-Lewis-McHale-Asif (DLMA) method. In the DLMA method, the model to estimate the resources is completely changed. For instance, the model for expected remaining runs as a function of overs left *u* and wickets lost *w* can be written as

$$Z_{ma}(u,w|\lambda )=Z_{0}\lambda^{n(w)+1}F(w)\left\{\frac{{tan}^{-1}(\frac{u-\mu_{0}}{\theta_{0}\lambda^{n(w)}F(w)})-{tan}^{-1}(\frac{-\mu_{0}}{\theta_{0}\lambda^{n(w)}F(w)})}{\frac{\pi }{2}-{tan}^{-1}(\frac{-\mu_{0}}{\theta_{0}})}\right\}$$ (2)

$$where\;F(w)=\frac{\Phi (10;\mu_{1},\theta_{1})-\Phi (w;\mu_{1},\theta_{1})}{\Phi (10;\mu_{1},\theta_{1})-\Phi (0;\mu_{1},\theta_{1})},$$

The function Φ(*w*; *μ*_1_, *θ*_1_) is a normal cumulative distribution with location *μ*_1_ and scale *θ*_1_. Further, *Z*_0_ and λ may be interpreted similarly as that of in the DLPro, the *μ*_*0*_ (≤0) and *θ*_0_ (>0) are the location and scale parameters to be estimated and *n*(*w*) = α+β*F*(*w*). The resources remaining can be mathematically written as

$$P_{ma}(u,w|\lambda )=\lambda^{n(w)-n(0)}F(w)\left\{\frac{{tan}^{-1}(\frac{u-\mu_{0}}{\theta_{0}\lambda^{n(w)}F(w)})-{tan}^{-1}(\frac{-\mu_{0}}{\theta_{0}\lambda^{n(w)}F(w)})}{{tan}^{-1}(\frac{N-\mu_{0}}{\theta_{0}\lambda^{n(0)}})-{tan}^{-1}(\frac{-\mu_{0}}{\theta_{0}\lambda^{n(0)}})}\right\}$$ (3)

where *N* is the number of pre-allotted overs to each two competing teams. It is noticeable that a total of six estimated parameters are required to calculate the remaining resources at any stage of the innings. Apart from developing alternative models, McHale and Asif [10] also proposed to use a direct scale in all cases. Thus they used *T* = *SR*_2_/*R*_1_ if *R*_2_≤*R*_1_ or *R*_2_>*R*_1_, where *T* is the revised target for the team batting in the second innings (Team 2)

### d. The Stern version of the DL method

Stern [2] proposed a modified form of the DLPro method. The method is referred as Duckworth-Lewis-Stern (DLS) method. In DLS, the model of the DLPro method remained the same except modification in the parameter λ (the match factor). He argued that the exponential decay should be adjusted according to the situation. Therefore, he suggested function *g* to adjust parameter *b* throughout the innings. The DLS model mathematically may be written as

$$Z_{dls}(u,w|\lambda )=Z_{0}\lambda^{n(w)+1}F(w)\left[1-\exp\left\{-ubg(u,\lambda )/\lambda^{n(w)}F(w)\right\}\right]$$ (4)

$$where,\;g(u,\lambda )={\left(\frac{u}{\lambda }\right)}^{-(1+\alpha_{\lambda }+\beta_{\lambda })}$$

with $\alpha_{\lambda }=-1/\left\{1+c_{1}(\lambda -1)e^{-c_{2}(\lambda -1)}\right\}$, and $\beta_{\lambda }=-c_{3}(\lambda -1)e^{-c_{4}(\lambda -1)}$. Furthermore, *F*(*w*) = 1+α_1_*w+* α_2_*w*^*2*^*+* α_3_*w*^*3*^_._ The functional form for *n*(*w*) was missing in Stern [2]. The resources remaining can be written mathematically as

$$P_{dls}(u,w|\lambda )=\lambda^{n(w)-n(0)}F(w)\left\{\frac{1-exp\{-ubg(u,\lambda )/\lambda^{n(w)}F(w)\}}{1-exp\{-Nb/\lambda^{n(0)}\}}\right\}$$ (5)

The resource function requires 9 + *x* number of parameters to be estimated, where *x* is the number of parameters in function *n(w)*. Stern [2] claimed that the DLS model is more flexible; however, it is not clear how much flexibility captures the different strategies of the runs scoring pattern of the game and does not violate the game’s essential properties cricket.

## 3 Comparative study of DLS and DLMA

*P(u*,*w)* provides the remaining resources at overs *u* left, and wicket lost *w*. Therefore, *Q(v*,*w) = 1—P(u*,*w)* are the resources used in *v = N—u* overs played and wicket lost *w*. Strictly speaking, *Q(v*,*w)* is the proportion of runs scored in overs *v* such that wicket *w* has already been lost in *N* overs match. The first-order derivative *∂/∂v Q*(*v*,*w*) *= ∂/∂u P*(*u*,*w*) as *∂v = -∂u* is the rate of change of resources used and may be considered as runs scoring potential on the current ball. The curve of *∂/∂v Q*(*v*,*w*) will depict the ball-by-ball resources and may be considered as a run-scoring pattern. Moreover, the second-order derivative *∂*^*2*^*/∂v*^*2*^ *Q*(*v*,*w*) = -*∂*^*2*^*/∂u*^*2*^ *P*(*u*,*w*) is the rate of change of ball-by-ball resources. The value of *v* that maximizes *∂*^*2*^*/∂v*^*2*^ *Q*(*v*,*w*) is the inflection point in the curve that depicts the ball-by-ball resources.

### a. Flexibility

It is desirable in the model-building to have flexibility towards the important features of the game of cricket. For example, an exciting part of the DLMA method is the flexibility to capture different runs scoring patterns of the LOI cricket, as may be depicted by the curve *∂/∂v Q*(*v*,*w*). For example, three black curves (solid, dotted, and dashed lines) in Fig 1 are the varieties of runs scoring patterns, as determined using DLMA for a variety of values of *μ* and *θ* given that *w = 0*, *λ* = 1, and *N = 50*. For example, the solid black line is for *μ = 0*, and *θ* = 22.7 that depict a common runs scoring pattern (over-by-over resource value) of the LOI, in which during the early stage of an innings, the batsmen increase the speed of scoring proportion of runs with respect to the progression of innings (overs played, *v = N—u*) with increasing acceleration (rate of change of over-by-over resources) provided that the wicket has not been lost. However, since there are finite runs to be scored on a ball, at certain point batters, runs-per-ball may not increase (of course, non-decreasing) with increasing acceleration with respect to progression of the innings (overs played). In Fig 1, the inflection point may be spotted at *v = 36* (or *u = 14*), in that the curve changes from concave to the convex.

In contrast, the DLS for *λ = 1*, *N = 50*, and any value of *b > 0*, the speed of scoring runs increases with increasing acceleration until the innings’ final ball with a given number of wickets lost, even for no wickets lost. Further, it is noticeable that *g(u*,1*) =* 1; hence, the parameters $c1, c2, c3, and c4 in the DLS has no role in the average or below run-scoring situation. Hence, in the DLS, for a given number of wickets lost, the runs scoring potential increases exponentially with respect to the progression of the innings. The blue curve in Fig 1 is the plot of ∂/∂*v Q*_dls_ for given *b = 0*.*043*, *N = 50*, *λ* = 1, with respect to the progression of the innings (*v*, overs played) that demonstrates the ball-by-ball resource value for the DLS.

Interestingly, the DLMA is flexible enough to approximate the run-scoring pattern similar to that of DLS; however, the converse is not true. For example, the black dashed line in Fig 1 is the run-scoring pattern of the DLMA, which is, to a greater extent, is similar to the blue curve of DLS. On the other hand, the DLS is not flexible enough to capture the runs scoring patterns of the DLMA; for example, black solid and dotted lines of the DLMA in Fig 1 cannot be approximated by DLS. Moreover, compared to RR method, the DLMA is flexible enough to capture the run-scoring pattern similar to that of the RR method. Here also, the converse is not valid. The red curve in Fig 1 shows the RR method’s run-scoring pattern, while the green line shows the DLMA for some suitable values of the model parameter values.

Considering another aspect of the flexibility, Stern [2] pointed out that in the DLPro, the resource value of the last ball is the same irrespective of the number of wickets lost as the derivative of the *Z* is independent of *w* at *u = 0*. Regarding this point, his comment is as “..*more wickets downs generally means poorer batsmen are more likely to be on strike*, *but this is by no means guaranteed and is likely a second-order effect”*. Hence, in any incarnation of the DL method, the resource value remains the same for both no-wicket lost and nine-wickets lost, which may be counter-intuitive.

It may be debatable whether the runs scoring potential of the innings’ final ball should be independent of the number of wickets lost. However, we believe that the model should be flexible enough to account for such an essential feature of the game. For example, in the DLMA, the parameter *μ*_0_ plays a vital role in accounting for the resource value on the last ball. For instance, *μ*_0_ = 0 indicates the resource value on the last ball is independent of *w*. The greater the absolute value of *μ*_0,_ show high the dependency on *w*. To illustrate, consider the derivative of the resources remaining with respect to *u*. The first derivative of *P* with respect to *u* is the measure of runs scoring potential (resource value) at *u = u*_*0*_.


$$\frac{\partial }{\partial u}P_{ma}(u,w|\lambda )=\frac{{F(w)\lambda }^{n(w)-n(0)}}{{tan}^{-1}(\frac{N-\mu_{0}}{\theta_{0}\lambda^{n(0)}})-{tan}^{-1}(\frac{-\mu_{0}}{\theta_{0}\lambda^{n(0)}})}\frac{\partial }{\partial u}{tan}^{-1}\left(\frac{u-\mu_{0}}{\theta_{0}\lambda^{n(w)}F(w)}\right)$$

$$=\frac{{F(w)\lambda }^{n(w)-n(0)}}{{tan}^{-1}(\frac{N-\mu_{0}}{\theta_{0}\lambda^{n(0)}})-{tan}^{-1}(\frac{-\mu_{0}}{\theta_{0}\lambda^{n(0)}})}{\left\{1+{\left(\frac{u-\mu_{0}}{\theta_{0}\lambda^{n(w)}F(w)}\right)}^{2}\right\}}^{-1}(\frac{-\mu_{0}}{\theta_{0}\lambda^{n(w)}F(w)}),$$

Further simplifications yield

$$\frac{\partial }{\partial u}P_{ma}(u,w|\lambda )=C_{N}{\left\{1+{\left(\frac{u-\mu_{0}}{\theta_{0}\lambda^{n(w)}F(w)}\right)}^{2}\right\}}^{-1}$$ (6)

where, $C_{N}=\frac{1}{\theta_{0}\lambda^{n(0)}}{\left\{{tan}^{-1}(\frac{N-\mu_{0}}{\theta_{0}\lambda^{n(0)}})-{tan}^{-1}(\frac{-\mu_{0}}{\theta_{0}\lambda^{n(0)}})\right\}}^{-1}$ is the function of constant terms.

From Eq (6) it is clear that the derivative of resources remaining is not independent of *w* for any value of *u*≥0. However, if *μ*_0_ = 0, then ∂/∂u *P*_ma_ is independent of *w* for *u = 0*. The large magnitude of the estimated value of *μ*_*0*_ indicates greater differentials in the resource value for different wickets lost on the last ball of the innings.

**Fig 2:**
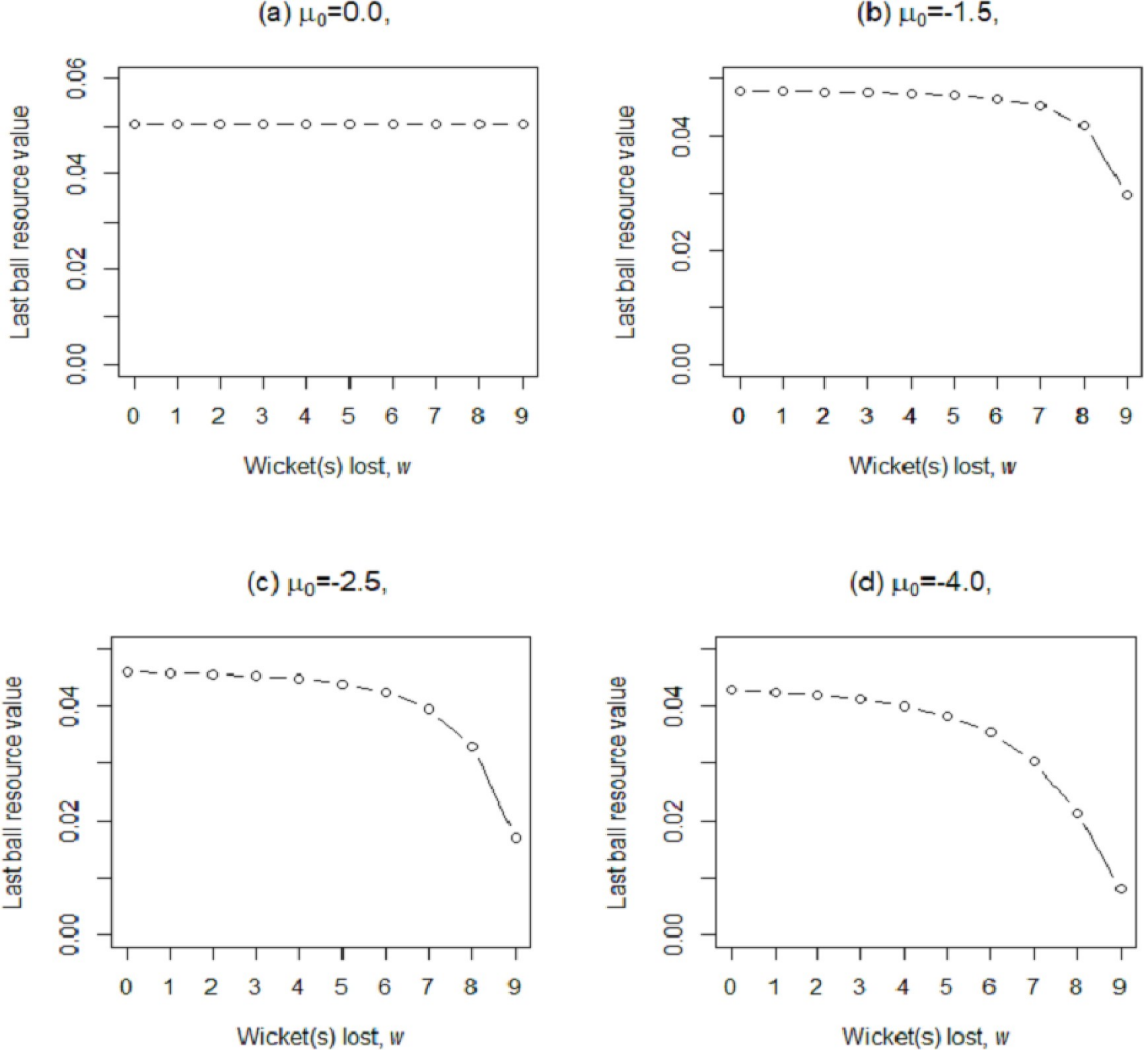
Plots of last ball resource value vs wickets lost (*w*) for four different values of *μ*_*0*_.

This concept for DLMA may better be demonstrated by plotting the last ball resource value against the number of wickets lost, *w*, for various estimated values of the *μ*_0_. Fig 2 shows four plots of ∂/∂*u P*_ma_ against *w*, at *u = 0* for *μ*_0_ = 0.0, -1.5, -2.5, -4.0. Fig 2A shows equal resource values for all wickets provided that the estimated value of *μ*_0_ = 0.0. In contrast, the last ball resource value is a decreasing function of *w* for all *μ*_0_ <0.0 (note that while estimating parameters, *μ*_0 is_ restricted to be less than or equal to zero). It is interesting to note that for a non-zero value of *μ*_*0*,_ the decay is rapid for the last few wickets compared to the top-order wickets. For example, in Fig 2B, the difference in resource value for the last couple of wickets is reasonably larger than top-order wickets. This is intuitive as it is highly likely that more wickets down means lower quality batters are on strike. From Fig 2, it is clear that a smaller value of *μ*_*0*_ (≤0) implies high differentials in the resource values of different numbers of wickets lost. McHale and Asif [10] estimated *μ*_*0*_ *= -1*.*33* for ODI cricket.

Hence, for this aspect again, the DLMA method is more flexible than the DLS. In the DLS, last ball resource values are independent of wickets lost for any estimated parameters value.

### b. Intuition

The McHale-Asif model for the DL method is not counter-intuitive for any estimated value of the parameters in the given range as the model is developed based on the four essential properties (See Asif [40] p.24), and McHale and Asif [7]). The DLS method provides counter-intuitive runs scoring patterns for the verity of estimated values of c1, c2, c3, and c4. This is especially evident in well above runs scoring matches. Since, Stern [2] did not provide enough information regarding the estimates. Therefore, we experimented on different possible values of c1, c2, c3, and c4 for given *N = 50*, *λ = 1*.*25*, *b = 0*.*045*, *and w = 0*. Similar, anomalies can be observed for other values of *N*, *b*, *λ>1*, *0≤w<10*.

**Fig 3:**
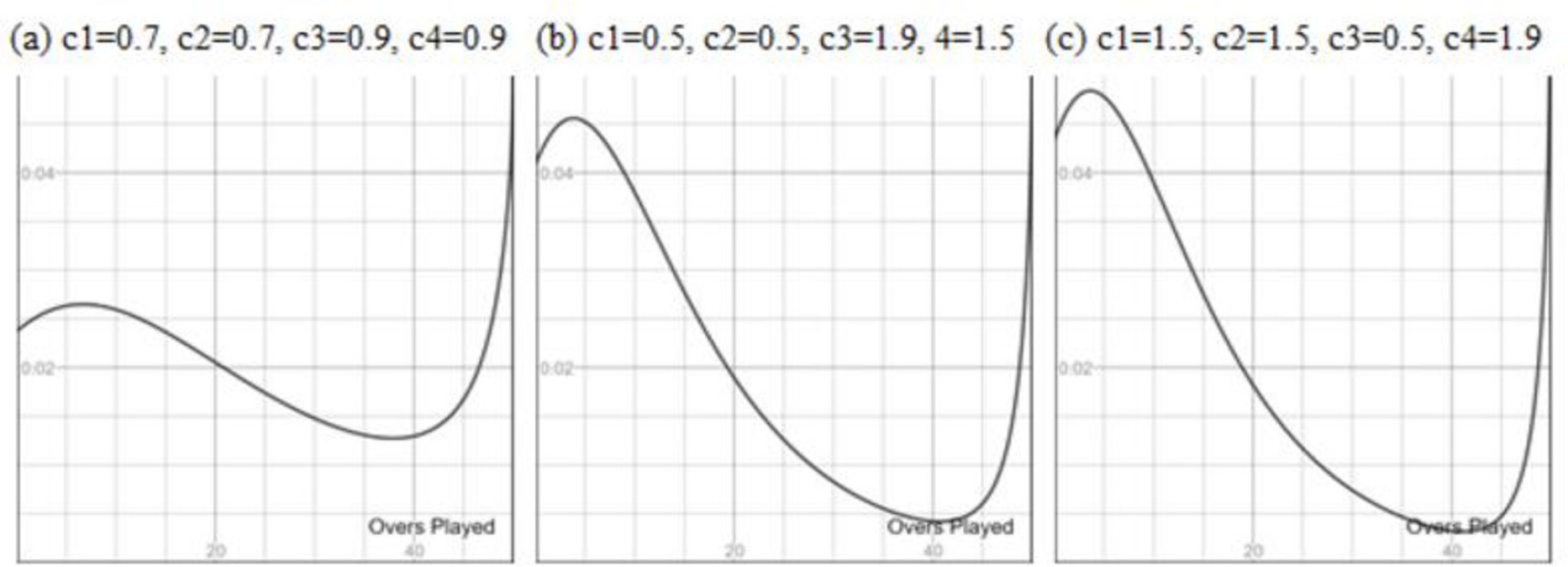
The ball-by-ball resources (y-axis) for zero-wicket lost with respect to the Overs Played in One-Day cricket.

To examine the runs scoring pattern, we developed a dynamic plot for ∂/∂*v* Q_dls_ with slides c1, c2, c3, and c4. Fig 3 is the graphical demonstration of the ball-by-ball resource value (a runs scoring potential) as obtained using DLS for the various values of c1, c2, c3, and c4.

The visual inspection of Fig 3 clearly shows that using the DLS method resource value of the next ball decreases with respect to the progression of the innings provided no-wicket lost. This is the counter-intuitive run-scoring pattern of LOI cricket. Similar anomalies may be observed for more wickets lost.

**Fig 4:**
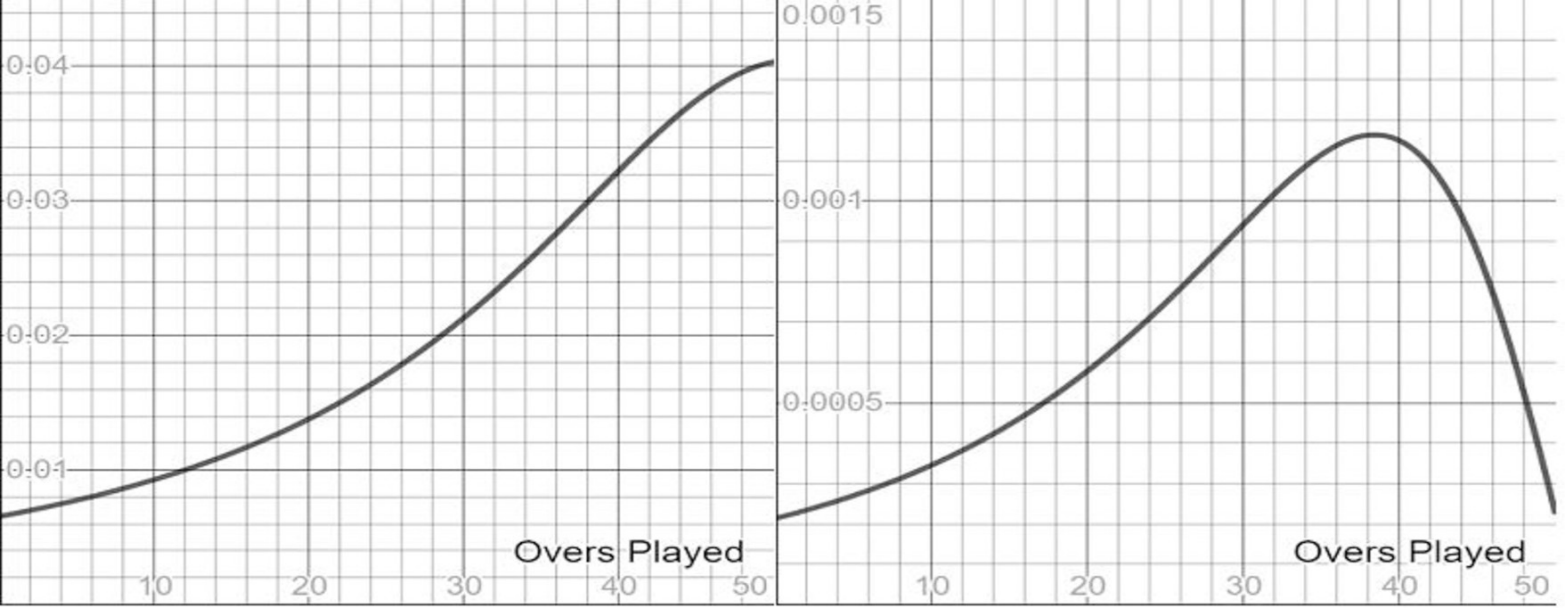
Plots for DLMA depicting the rate of change of resources used (left panel) and rate of change of over-by-over resources (right panel).

Fig 4 shows the run-scoring pattern of the fitted DLMA. There is a point of inflection at approximately 38 overs played, given that no wicket lost. This is again intuitive to the nature of the limited-overs game of cricket. As a result, after the inflection point, the acceleration (as depicted in Fig 4 (right panel) of ball-by-ball runs is declined with respect to the progression of the innings. In contrast, the DLPro has no point of inflection, while Stern [2] reported that DLS adjusts the rate of change according to the scenario. However, no explanation of adjustment to the scenario was provided. Further, as no information regarding the estimates and estimation procedure of c1, c2, c3, and c4 are provided for DLS, therefore, similar plots cannot be generated for DLS.

### c. Simplicity and goodness of fit

The DLMA method is not only more ‘flexible’ and intuitive as compared to the DLS, but also the DLMA is a comparatively simple model as; firstly, it requires less number of parameters to be estimated with better goodness of fit [10]. Secondly, it is simple to understand how the ball-by-ball resource curves (the runs scoring pattern) vary by changing the estimates’ values. For example, the larger value for *θ*_0_ shows more uniformity in the ball-by-ball runs scoring potential, and the point of inflection tends to settle at a larger value of *u* (i.e., during the earlier stage of the innings). For example, compare the two black curves (solid and dotted lines) in Fig 1. The value of *θ*_0_ for the dotted line curve is greater than the value of *θ*_0_ for the solid line curve of DLMA. Similarly, the effect of changing values of *μ*_0_, already discussed in section 3b.

**Fig 5:**
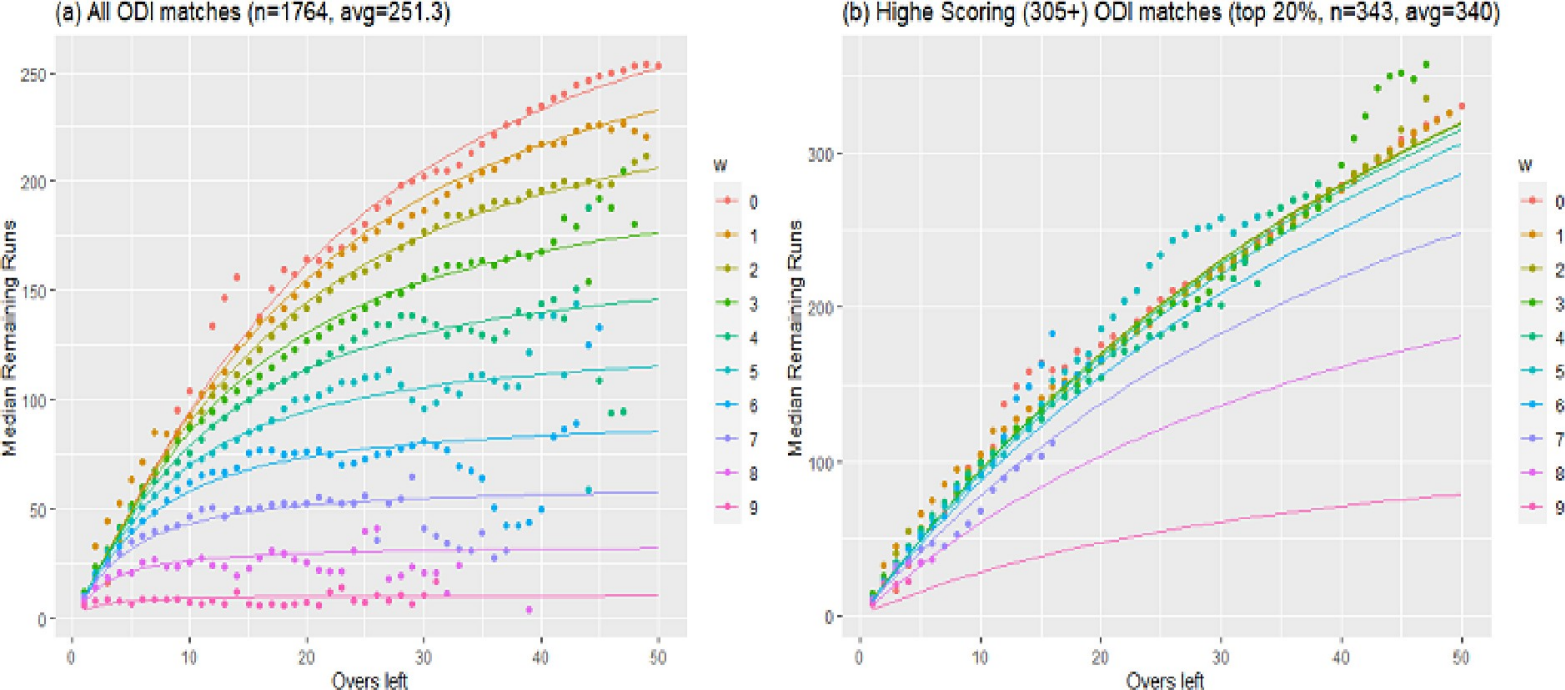
DLMA model fit on median remaining runs for the data of 1764 uninterrupted first innings One-Day International (ODI) cricket.

Regarding the goodness of fit, McHale and Asif [10] reported that the DLMA was better fitted to the data than the DLPro using the then-available data for the limited number of matches. To investigate the goodness of fit of both models, we used the updated data for the uninterrupted 1764 ODI matches played from Jan-2004 to Jun-2021. The ball-by-ball of these matches were obtained from the website https://cricsheet.org/. We fit both models using R software. The results show that both models are equally efficient using the overall ODI data. However, for well above runs scoring matches, the DLMA has a better fit for the data. This argument is in-line with the Stern [2] report that DLPro is incompatible with the well above runs scoring matches, and therefore, proposed the DLS method by modifying the DLPro method. He argued that the resources (or remaining runs) should be more linear in high runs scoring matches. However, as discussed above, such modification made the DL method counter-intuitive. Fig 5 shows the DLMA fit for all data of 1764 ODI matches and for the data with well-above or high scoring matches (i.e. all those first innings where the total runs were more than 305, top 20%). It can be seen in Fig 5B that the median remaining runs as a function of overs left are considerably more linear as compared to the plot in Fig 5A.

### d. Compatibility with Twenty-20 cricket

After introducing the Twenty-20 International cricket in 2005, fans and cricketing authorities were curious whether the DL method, designed for ODI cricket, is compatible with the Twenty-20 cricket. However, the debate was highlighted on media after the two controversial applications of the DLPro method in the ICC world Twenty-20 in May-2010. First, West Indies won against England’s 191 in the first inning just by scoring 60/2 in six overs. Second, Sri Lanka close to being eliminated as Zimbabwe were only required 44 runs in five overs in response to Sri Lanka’s 173 in the first innings.

Consequently, McHale and Asif [10] suggested using a single model for ODI and T20I. Analyzing and comparing the data of ODI and T20I, they concluded that there was no evidence of statistically significant differences observed so that a separate model should be used. Specifically, they performed a series of independent t-tests to test the significance of the difference in mean remaining runs at each overs remaining (ranging from 20 to 1) for each value of *w* (ranging from 0 to 9) for T20I and ODI. Performing 94 independent t-tests produced just three statistically significant differences in means at the 5% level.

Similarly, Stern [2] also analyzed the data of the two formats, and the results were consistently similar to that of McHale and Asif [10]. Moreover, Asif and McHale [7] developed a model for T20I using the GNLFM to estimate the runs margin of victory for the winning team batting in the second innings. The model specification was similar to DLMA developed by McHale and Asif [10] using the combined data of ODI and T20I. Hence, it is concluded that the DLMA may safely be used for Twenty-20 cricket.

## 4 Conclusion

In this paper, a comparative study was done to examine the suitability and relative efficiency of the DLS and DLMA methods for resetting targets for the team batting in the second innings in an interrupted LOI cricket match. The research findings have shown that DLMA is intuitively more flexible, simpler, and better fit to the data than the DLS method.

The flexibility of the two methods is evaluated by visualization of the run-scoring pattern. It was evident in Fig 1 that DLMA is flexible enough to capture the runs scoring pattern as depicted by DLS. However, the converse is not true. In the DLMA, an additional exciting feature is accounting for the last ball resources dependency on the number of wickets lost. For instance, in the DLMA, the resources on the innings’ last ball are independent of the number of wickets lost only if *μ*_*0*_ *= 0*. In contrast, the DLS method always considers the last ball resources of the innings independent of the number of wickets lost. Hence, it is concluded that DLMA is more flexible as compared to the DLS method.

In regard to examining the intuition of the two methods, it was shown that DLMA does not violate any basic principle of the game as described in Stern [2], Asif and McHale [7] and Asif [40]. However, in DLS, for a variety of values of c1, c2, c3, and c4 the run-scoring pattern was counter-intuitive, as demonstrated in Fig 2. Moreover, it is argued that DLMA is comparatively simple. Firstly, it requires considerably fewer parameters to be estimated with a better fit to the data. Secondly, DLMA is simple to understand the variation in curves by changing the values of estimated parameters.
